# Market Prices

**Published:** 1885-04

**Authors:** Thos. K. Beecher


### MARKET PRICES.

THOS. K. BEECHER.

The American Encyclopedia (*sub voce* "DOLLAR") states, as I suppose accurately, facts as follows: —

"The dollar-unit . . . was established by "act of congress of April 2, 1792. The silver "dollar was first coined in 1794 weighing 416 "grains . . . 892.4-thousandths of which was "pure silver." In 1837 the weight of this coin was reduced to 412½ grains, in which, however, "the quantity of pure silver remain- "ing 371¼ grains as before" the *bullion value* of the coin was unchanged. The loss of weight 3½ grains was due to the less alloy. This silver dollar was and is legal tender.

In 1849 (Act March 3) gold dollars also were coined, containing 23.22 grains of pure gold, the coin weighing 25.8 grains, one-tenth of the total weight being alloy.

Here then we have the "dollar unit" expressed by a silver coin and also by a gold coin—in both cases by laws of equal authority. These coins are, the one a bit of silver, the other a bit of gold—both of them being *metals* that are bought and sold in the market by the *dwt.*, *oz.* and *lb.*—Troy weight.

When bits of metal are punched and stamped according to law, they become "dollars." Before they are punched they are sheet metal—bullion—bought and sold by the *dwt.* ("pennyweight.") When punched but not stamped they are "buttons" or "blanks." When stamped they are coins—dollars—and are endued with the power of paying debts—legal tender power.

A farmer cannot pay his debts with wheat, nor with butter, nor with live-stock, except he and his creditors can agree upon the bargain or swop. But he can pay his debt and compel his creditors to accept a certain number of metal dollars; or the creditor may exact a certain number of metal dollars.

That is to say, congress obeying the so-called "laws of trade and finance" has enacted that a certain weight of certain metals, shall at all times and in every place be called a dollar, and be worth a dollar in paying debts. Not every metal, but only silver and gold!

Were congress to enact that one net ton of bar iron, refined, and three-times worked under the hammer, shall be called sixty dollars, the men of commerce would probably object. Congress has no power to fix the price of iron! If Congress were to enact that five pounds of copper is a dollar, there would be another howl of derision and protest! Should congress persist, and enact that a pound of butter nine-tenths butter, and one-tenth water and salt is *twenty-five cents*, and a legal tender in payment of debt at that rate—400 pounds of butter 100 dollars—idiotic! absurd! stark lunacy!

In short there is not a commodity that the skill and labor of man can produce and send to market to which congress can affix a value except the two metals gold and silver. Ten thousand things that men buy and sell at market rates, and yet only two things—silver and gold—that must be bought and sold at prices fixed by law!

Ten different ores dug from the mines, and smelted into metals—Iron, Lead, Tin, Zinc, Copper, Nickel, Aluminum, Silver, Platinum and Gold—sell them in open market for what you can get—all of them *except* silver and gold. But these shall be worth as follow; 371¼ grains of pure silver shall fetch a dollar, and 23.22 grains of pure gold shall also fetch a dollar!

I ask this question: Is it reasonable or just for the resistless power of law to fix the debt-paying power of two metals, at one constant value in "dollars," and leave the debt-paying power of all other commodities, to take the chances of the market—leaving debtors to the mere whim of the creditor.

But I hear it said, that we must agree upon some "standard of value" a "monetary yard stick." We must have something that does not fluctuate; something constant; we must have "*an honest dollar*," "a hundred cents in every dollar." "Gold and silver are our standards."

Again I ask: Here are ten metals; Eight of them fluctuate so widely in value that it were monstrous absurdity for congress to attempt to fix their value by law! But oh wonder! here are two metals that do not fluctuate!—gold and silver! They are our standards. Is not such a claim *prima facie* questionable if not absurd?

Once upon a time two railway conductors compared watches. They varied fifteen minutes. Said one "mine is a *standard* Waltham watch warranted." Said the other—"Mine is an Elgin watch warranted." Said the first, "mine is *standard*—I received it from the

Company, we must have a standard." So also said the other. One was a gold-cased watch, the other was a silver-cased watch and both were *standards.* The controversy waxed warm. The "*standard*" watches differed 15 minutes just as our "*standard*" dollars differ fifteen cents.

A bystander might suggest to the conductors that perhaps both "standards" were wrong; and that smashing one watch will not prove that the other one is right on "time by the stars." "Stars"! they both sneer, "stars! run trains by stars"! To whom the bystander might reply, "Gentlemen, no one watch made by man can ever be safely trusted as a standard of time. If you wish to make close connections with railways that belt a continent, you or some wiser man than you must consult the stars." Which thing railways in the matter of time have learned to do with profit.

But congress and practical men of trade have not yet learned that no one metal can be honestly erected as a standard of value, and its debt-paying value fixed by law without involving monstrous absurdity, oppression, cruelty and commercial wreck, as it is this day.

Fortunately for popular education congress has endowed with equal debt-paying power the silver aud the gold dollar. Both dollars are equally "honest," or, as I prefer to say, EQUALLY DISHONEST. A child can see that the standards disagree by 15 cents. Whether the silver be over-valued, or the gold under-valued, or both of them falsely valued who is ready to settle *authoritatively?* Smashing one will not prove the other just and honest! Let the controversy go on, until the clamor shall have wakened us all to see the enormous stupidity of calling either one a *standard:*—The enormous injustice of requiring debts to be paid in one and but one commodity—*metal;* and among metals promoting but two to this high function of debt-paying between man and man!

The time will yet come, when all men will see clearly that gold and silver are *metals;* and like all other metals should be bought and sold in open market, for what they will bring in free exchange for other commodities. And that when governments exalt these two metals to the royal function of money, the enactment does actually create a monopoly, compared with which all other injustices and cruelties that attend upon commercial warfare are feeble and pale.

